# Correction: Assessment of the transmission blocking activity of antimalarial compounds by membrane feeding assays using natural *Plasmodium falciparum* gametocyte isolates from West-Africa

**DOI:** 10.1371/journal.pone.0315144

**Published:** 2024-12-03

**Authors:** Noëlie B. Henry, Issiaka Soulama, Samuel S. Sermé, Judith M. Bolscher, Tonnie T. G. Huijs, Aboubacar S. Coulibaly, Salif Sombié, Nicolas Ouédraogo, Amidou Diarra, Soumanaba Zongo, Wamdaogo M. Guelbéogo, Issa Nébié, Sodiomon B. Sirima, Alfred B. Tiono, Alano Pietro, Katharine A. Collins, Koen J. Dechering, Teun Bousema

Figs 3, 4 and 5 are uploaded incorrectly. Please see the correct Figs and associated captions here.

**Fig 3:**
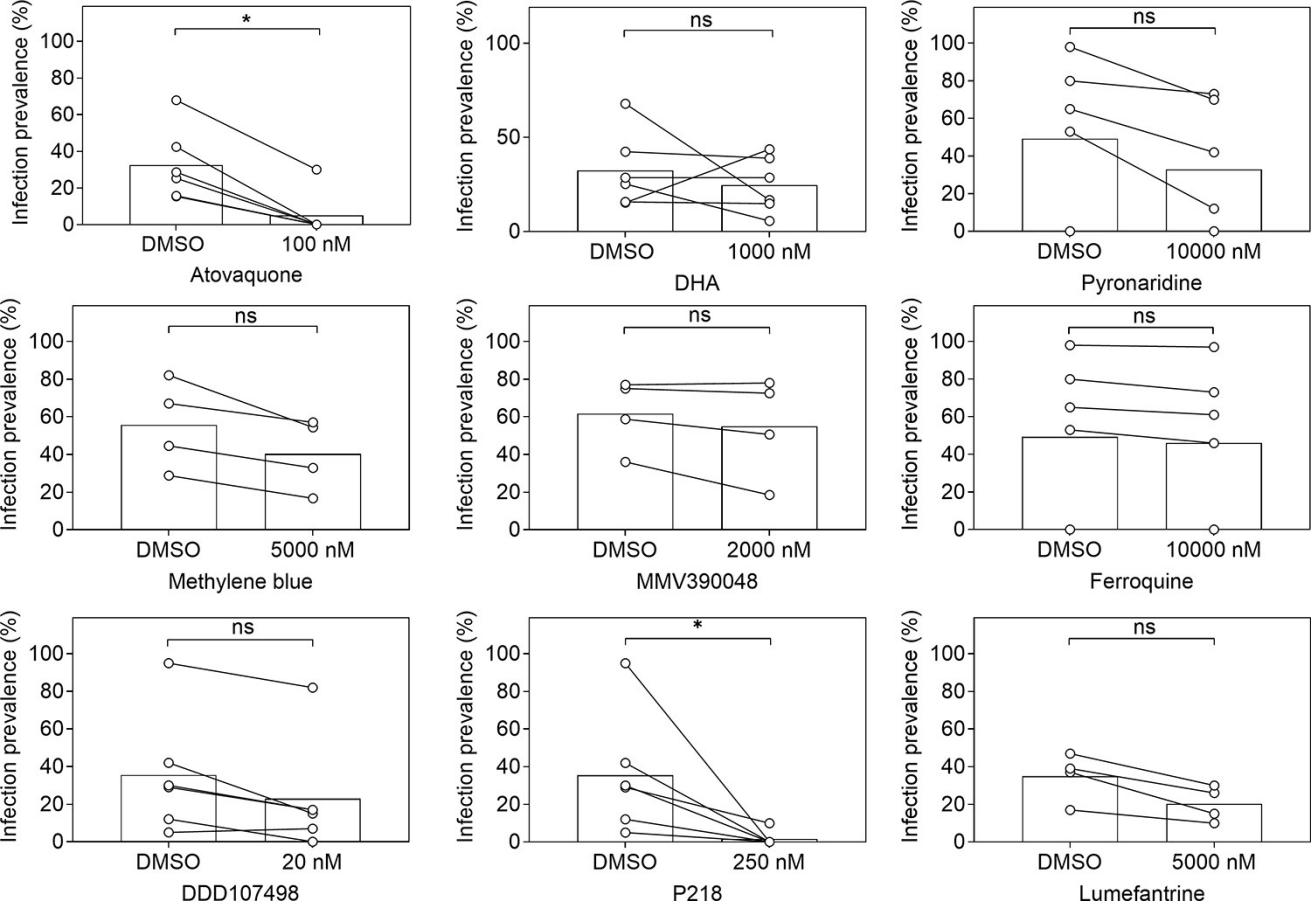
Transmission-blocking effects of compounds when directly added to Plasmodium falciparum gametocyte field isolates prior to feeding. Effects of Atovaquone, DHA, Pyronaridine, Methylene blue, MMV390048, Ferroquine, DDD107498, P218 and Lumefantrine on the proportion of mosquitoes that became infected after feeding on gametocytes from naturally infected gametocyte donors where serum was replaced and the compound was added to the blood meal immediately prior to feeding. Every symbol represents an individual gametocyte donor whose blood was offered to mosquitoes with DMSO control (0.1% DMSO) or the compound at a single concentration. Lines connect experiments performed on the same blood aliquot; bars indicate the mean infection prevalence. The asterisks indicate statistical significance (p<0.05) in a Wilcoxon matched-pairs signed-rank test. Of note, the number of observations is small so lack of statistical significance should not be interpreted as evidence of no difference. Experiments with no infected mosquitoes in the DMSO control were not included in the statistical analyses.

**Fig 4:**
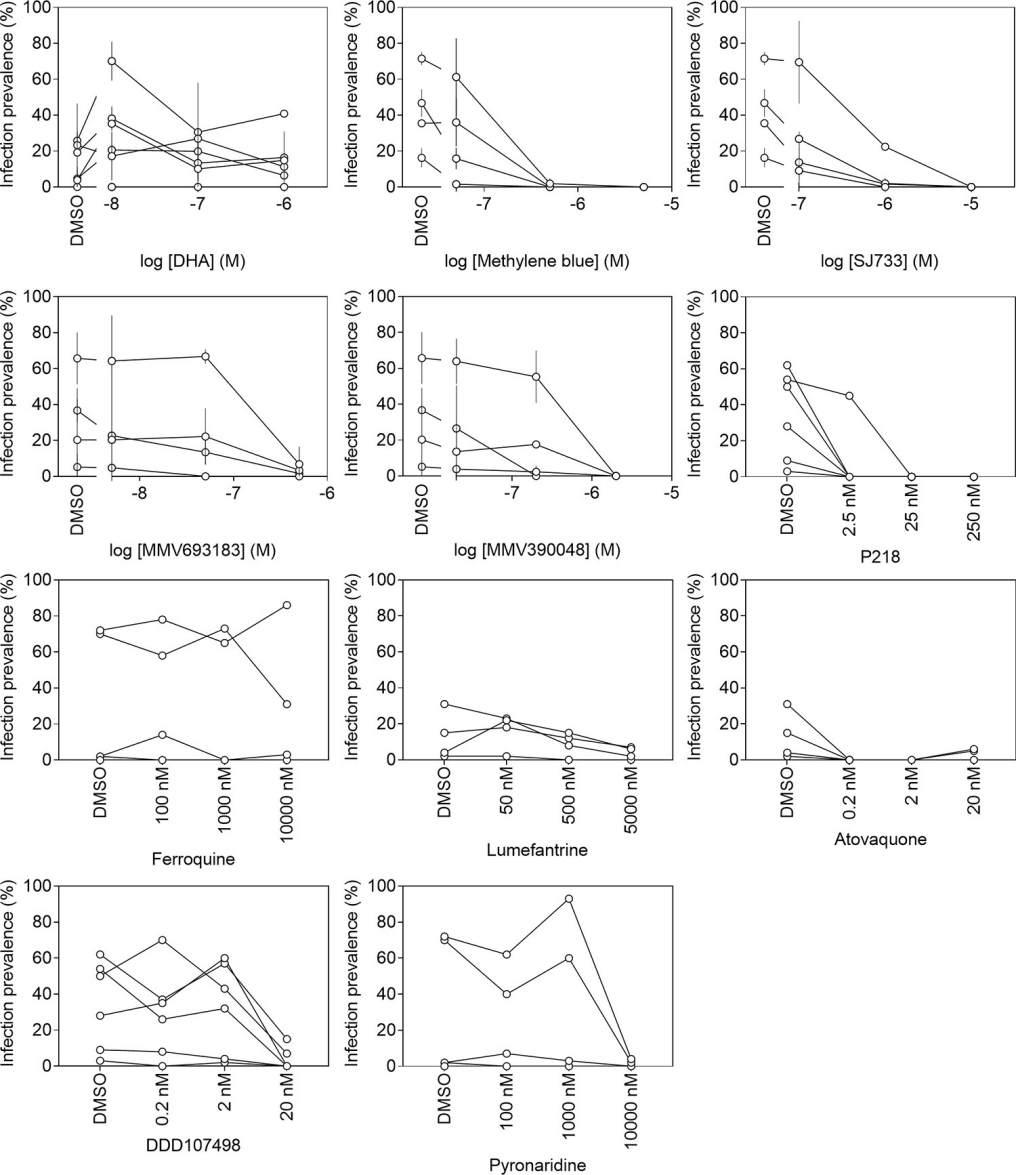
Transmission-blocking effects of compounds when incubated with Plasmodium falciparum gametocyte field isolates. Serial dilutions of compounds were added to gametocytes in RPMI/European serum A and incubated for 24 hours prior to feeding. The plot indicates the effects on mosquito infection prevalence of DHA, Methylene Blue, SJ733, MMV390048, MMV693183, DDD107498, P218, Pyronaridine, Ferroquine, Lumefantrine, and Atovaquone. DMSO was used as a negative control. Symbols indicate individual donors with connecting lines indicating the different incubation conditions for the same donor. Error bars indicate standard deviations from technical replicates (n = 2); some compounds were only tested in with a single technical replicate and therefore no error bars are provided.

**Fig 5:**
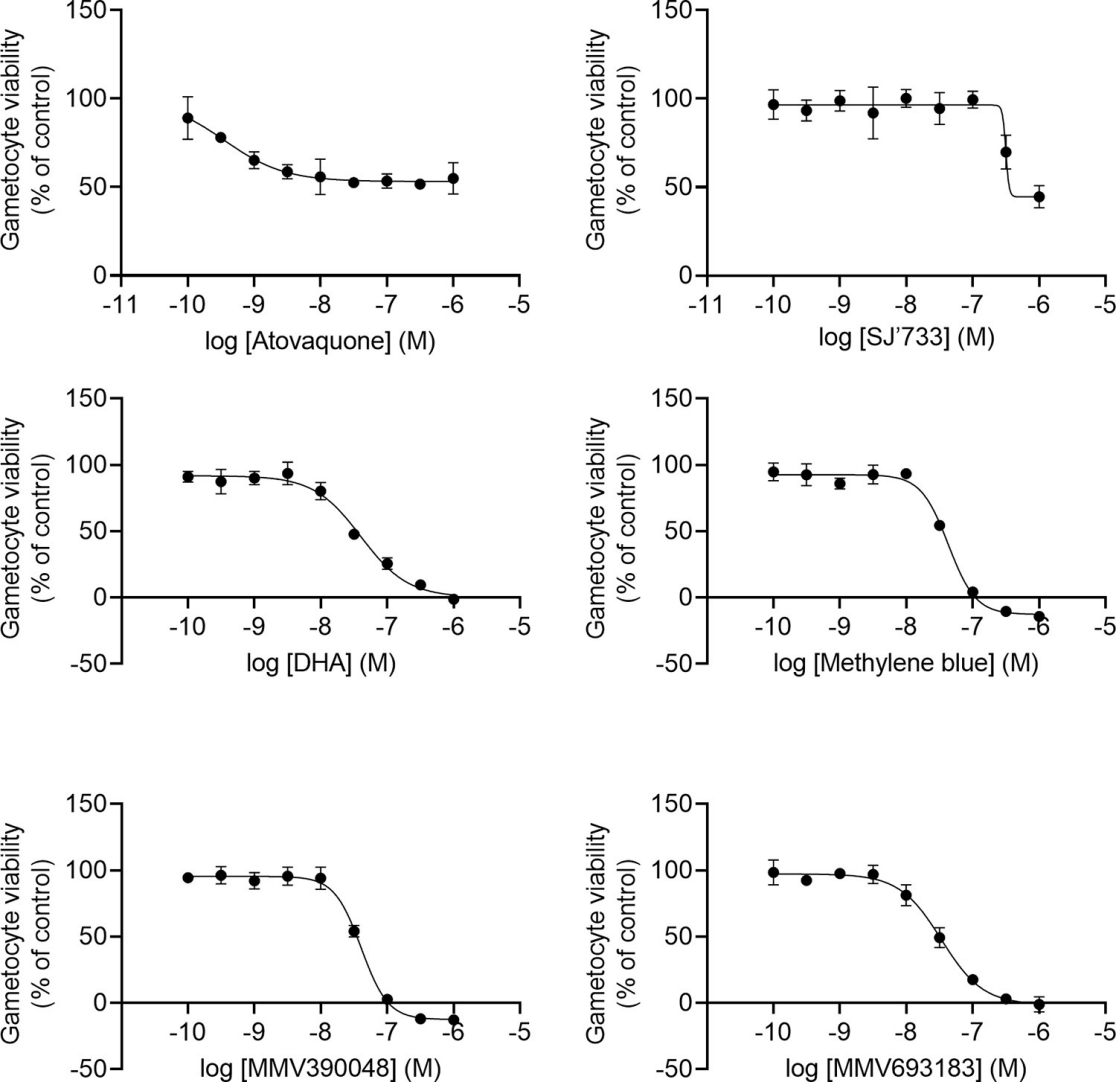
Effect of transmission-blocking compounds when incubated with cultured Plasmodium falciparum gametocytes. Serial dilutions of compounds were added to cultured gametocytes and incubated for 72 hours upon which gametocyte viability, determined by luminescence, was calculated at percentage of DMSO control. The graphs indicate the effect of DHA, Methylene blue, SJ733, MMV390048, MMV693183 and Atovaquone on gametocyte viability. Error bars indicate standard deviation. Error bars indicate standard deviations.
