# Supplementary material for: Assessment of the transmission blocking activity of antimalarial compounds by membrane feeding assays using natural Plasmodium falciparum gametocyte isolates from West-Africa
Source: PLoS One. 2023 Jul 26;18(7):e0284751. doi: 10.1371/journal.pone.0284751 (PMC10370769; doi:10.1371/journal.pone.0284751)
Supplement: Attachment — Submitted filename: Reviewers Comments_final.docx [file pone.0284751.s003.docx]

Journal Requirements:

Please review your reference list to ensure that it is complete and correct. If you have cited papers that have been retracted, please include the rationale for doing so in the manuscript text, or remove these references and replace them with relevant current references. Any changes to the reference list should be mentioned in the rebuttal letter that accompanies your revised manuscript. If you need to cite a retracted article, indicate the article’s retracted status in the References list and also include a citation and full reference for the retraction notice.

The reference list is complete. The reference number 7 was not cite correctly and modify tob e cited correctly; it is cited now as “WWARN Gametocyte Study Group. Gametocyte carriage in uncomplicated Plasmodium falciparum malaria following treatment with artemisinin combination therapy: a systematic review and meta-analysis of individual patient data. BMC Med. 2016 May 24;14:79. doi: 10.1186/s12916-016-0621-7. PMID: 27221542; PMCID: PMC4879753.”

[Note: HTML markup is below. Please do not edit.]

Reviewers' comments:

Reviewer's Responses to Questions

**Comments to the Author**

1. If the authors have adequately addressed your comments raised in a previous round of review and you feel that this manuscript is now acceptable for publication, you may indicate that here to bypass the “Comments to the Author” section, enter your conflict of interest statement in the “Confidential to Editor” section, and submit your "Accept" recommendation.

Reviewer #1: (No Response)

Reviewer #2: All comments have been addressed

2. Is the manuscript technically sound, and do the data support the conclusions?

The manuscript must describe a technically sound piece of scientific research with data that supports the conclusions. Experiments must have been conducted rigorously, with appropriate controls, replication, and sample sizes. The conclusions must be drawn appropriately based on the data presented.

Reviewer #1: Partly

Reviewer #2: Yes

3. Has the statistical analysis been performed appropriately and rigorously?

Reviewer #1: No

Reviewer #2: Yes

4. Have the authors made all data underlying the findings in their manuscript fully available?

The [PLOS Data policy](https://eur02.safelinks.protection.outlook.com/?url=http://www.plosone.org/static/policies.action%23sharing&data=05%7c01%7cteun.bousema%40radboudumc.nl%7c3d6d17d5db99491951f608db19df2168%7cb208fe69471e48c48d87025e9b9a157f%7c1%7c0%7c638132218368471660%7cUnknown%7cTWFpbGZsb3d8eyJWIjoiMC4wLjAwMDAiLCJQIjoiV2luMzIiLCJBTiI6Ik1haWwiLCJXVCI6Mn0%3D%7c3000%7c%7c%7c&sdata=vtET/7Jl3qHTPJvi%2BHQXzUeIsXDTdhM01ZmMBQvOfKU%3D&reserved=0) requires authors to make all data underlying the findings described in their manuscript fully available without restriction, with rare exception (please refer to the Data Availability Statement in the manuscript PDF file). The data should be provided as part of the manuscript or its supporting information, or deposited to a public repository. For example, in addition to summary statistics, the data points behind means, medians and variance measures should be available. If there are restrictions on publicly sharing data—e.g. participant privacy or use of data from a third party—those must be specified.

Reviewer #1: Yes

Reviewer #2: Yes

5. Is the manuscript presented in an intelligible fashion and written in standard English?

PLOS ONE does not copyedit accepted manuscripts, so the language in submitted articles must be clear, correct, and unambiguous. Any typographical or grammatical errors should be corrected at revision, so please note any specific errors here.

Reviewer #1: Yes

Reviewer #2: Yes

6. Review Comments to the Author

Please use the space provided to explain your answers to the questions above. You may also include additional comments for the author, including concerns about dual publication, research ethics, or publication ethics. (Please upload your review as an attachment if it exceeds 20,000 characters)

Reviewer #1: The authors replied to majority of my concerns appropriately, and clarity of the manuscript has been improved significantly. However, this reviewer thinks further minor modifications are required before publication.

1) Statements are not fully supported by the data
1-a) Fig 3 and interpretation of the results
First, data where zero prevalence in the DMSO control should be excluded from the statistical analysis (such as PYR with Donor 3 and 4), as we cannot tell whether there was not drug effect or not. Having said that, it is OK to include such data in the figure if the authors want to show how many assays had zero prevalence in the controls. Second, insignificant results by the Wilcoxon matched-pairs signed rank test are not interpreted appropriately (while the selection of the statistical test is reasonable). Based on the test, unless there are >5 pairs, the statistical results are always insignificant (i.e., even if each of all 5 paired data showed reduction from 100 to 0%, p=0.0625). The reason to see “significant” differences only in ATQ and P218 was because the two drugs were tested with 6 donors’ parasites. For MB, PYR, FQ and LUM, all donors’ parasites (4 out of 4) showed reductions in prevalence, so it is possible that the 4 drugs (at least some of them) could also show “significant” reductions if they were tested with 6 donors’ parasites. Line 303-307 and 422-425 should be rewritten, considering the limitation of the study design. In addition, “ns” in Fig 3 should be removed for drugs with <6 pairs. General readers think “ns” means no difference, instead of not enough statistical power.

RESPONS: We have addressed this. In the text, we have clearly indicated that caution is required in interpreting no significance as evidence of no difference. This has been indicated in the legend to figure 3 (page 12, line 317-318: ‘Of note, the number of observations is small so ns lack of statistical significance should not be interpreted as evidence of no difference’), in the Results section (lines 300-303 ‘These experiments showed that Atovaquone almost completely inhibited infectivity at 100nM and also demonstrated high potency of P218. With our limited number of paired feeds, we observed no evidence for a statistically significant effect of DHA, Methylene blue, MMV390048, DDD107498, Pyronaridine, Ferroquine, or Lumefantrine on gametocyte infectivity at micromolar concentrations (Figure 3)‘ and the Discussion section (line 424-426 ‘Although the number of gametocyte donors was modest and subtle effects cannot be ruled out, our data indicate … ‘

Of note, pairs with zero percent prevalence in the DMSO condition have been excluded from the statistical analysis. This has been clarified in the Methods and Figure legends. Also, the ‘ns’ has been removed from figure 3.

1-b) Correlations among the three assays (Table 1, Line 358-360, and Line 475-476)
It is reasonable to conclude that there was a significant correlation between SMFA and TB-DMFA (Spearman rank correlation coefficient of 0.92 with p=0.0004, excluding DHA data). But the conclusion written in Line 475-476 is opposite. A fair conclusion from this study is that there is a strong correlation between the two assays, except for DHA. Second, there is no correlation at all between TB-DMFA (or SMFA) and gametocyte viability assay by a Spearman rank test (p=0.777). Therefore, Line 358-360 seems incorrect, unless the authors checked the correlation differently (in such a case, please specific how it was tested).

RESPONS: We have rephrased this. ‘Comparison of IC50 values between gametocyte viability assay, TB-DMFA using field isolates and SMFA using NF54 laboratory strain shows broad agreement between the assays for Methylene Blue, MMV048, MMV693183, SJ733, Pyronaridine, DDD107498 and Lumefantrine while other compounds (notably DHA showed marked differences in activity against NF54 and field isolates.’ (line 359-363)

2) Table 1
Since comparing the three assays is the one of main point of this study, please add 95%CI of IC50 estimates, at least for TB-DMFA data. Showing the 95%CI helps readers to intuitively understand whether 38 (SMFA) and 167 (TB-DMFA) for MMV693183 are truly different, or within the error of

estimates, for example.

RESPONS: we appreciate this comment but, since we have tested only three concentrations for each compound the logistic regression model used to determine IC50 values did not converge to the point where it was able to resolve confidence intervals. We have refrained from strong statements about differences and have indicated the limitation of this study in the revised discussion section (“Our current study tested a limited set of concentrations per compound. Although this allowed a broad comparison between transmission-blocking effects observed against field isolates versus laboratory studies, a more detailed analyses of more subtle changes in potency would benefit from more extensive dose-response analyses.”).

In addition, please replace from “GCT-DMFA” to “TB-DMFA”
RESPONS: This has been addressed.

3) Exclusion of zero prevalence data from IC50 analysis (Fig 4)
Same as above point 1-a), such data should not be included for IC50 analysis. The authors might do so, but not written in the current text/figure/supplement

RESPONS: Experiments with zero prevalence in the DMSO controls were excluded from the IC50 analyses and this has been clarified in the legend to table 1. New analyses of the Pyronaridine data (without the zero prevalence experiment) led to a small change in the estimated IC50 from 8,754 to 8,640 nM

4) How to describe TB-DMFA
In Fig 1, please add “sporontocidal effect” for TB-DMFA (only “gametocyte effect” is written in the current figure). For clarity, at least in Line 326 and 345, please specify that the drug were added to the feeders as well. Readers, who do not read the method section carefully, could misunderstand that there was no drug in the blood samples which were fed to mosquitoes.

COMMENT: we appreciate this important rectification. We have updated this figure and legend (‘Natural gametocyte isolates were used for two distinct assays detecting the overall transmission-blocking activity of compounds by incubating them with gametocyte infected blood for 24 hours (2. TB DMFA; concurrently detecting both gametocyte and sporontocidal effects) or…’). We also updated the information in Lines 328 ‘This experimental set-up detects the effect of compounds against gametocytes and/or its sporontocidal effects’.

5) Replace (or take out) “overnight” incubation to “24 hour” incubation.
While the authors fixed the most of them, “overnight” are still seen in Line 39, 254 and 256

COMMENT: this has been updated.

Reviewer #2: (No Response)

7. PLOS authors have the option to publish the peer review history of their article ([what does this mean?](https://eur02.safelinks.protection.outlook.com/?url=https://journals.plos.org/plosone/s/editorial-and-peer-review-process%23loc-peer-review-history&data=05%7c01%7cteun.bousema%40radboudumc.nl%7c3d6d17d5db99491951f608db19df2168%7cb208fe69471e48c48d87025e9b9a157f%7c1%7c0%7c638132218368471660%7cUnknown%7cTWFpbGZsb3d8eyJWIjoiMC4wLjAwMDAiLCJQIjoiV2luMzIiLCJBTiI6Ik1haWwiLCJXVCI6Mn0%3D%7c3000%7c%7c%7c&sdata=Rz53PKsWw4Hb2iXNMn/gWZ8UQr1ehzQ5I/18sdeUqWc%3D&reserved=0)). If published, this will include your full peer review and any attached files.


If you choose “no”, your identity will remain anonymous but your review may still be made public.


**Do you want your identity to be public for this peer review?** For information about this choice, including consent withdrawal, please see our [Privacy Policy](https://eur02.safelinks.protection.outlook.com/?url=https://www.plos.org/privacy-policy&data=05%7c01%7cteun.bousema%40radboudumc.nl%7c3d6d17d5db99491951f608db19df2168%7cb208fe69471e48c48d87025e9b9a157f%7c1%7c0%7c638132218368471660%7cUnknown%7cTWFpbGZsb3d8eyJWIjoiMC4wLjAwMDAiLCJQIjoiV2luMzIiLCJBTiI6Ik1haWwiLCJXVCI6Mn0%3D%7c3000%7c%7c%7c&sdata=Lf6gQxNYQriMK3bXwkxz/ehxU4goY3DzawN9bNKIF%2BY%3D&reserved=0).

Reviewer #1: No

Reviewer #2: No

[NOTE: If reviewer comments were submitted as an attachment file, they will be attached to this email and accessible via the submission site. Please log into your account, locate the manuscript record, and check for the action link "View Attachments". If this link does not appear, there are no attachment files.]

While revising your submission, please upload your figure files to the Preflight Analysis and Conversion Engine (PACE) digital diagnostic tool, [https://pacev2.apexcovantage.com/](https://eur02.safelinks.protection.outlook.com/?url=https://pacev2.apexcovantage.com/&data=05%7c01%7cteun.bousema%40radboudumc.nl%7c3d6d17d5db99491951f608db19df2168%7cb208fe69471e48c48d87025e9b9a157f%7c1%7c0%7c638132218368471660%7cUnknown%7cTWFpbGZsb3d8eyJWIjoiMC4wLjAwMDAiLCJQIjoiV2luMzIiLCJBTiI6Ik1haWwiLCJXVCI6Mn0%3D%7c3000%7c%7c%7c&sdata=WhhF0yC7CwkzgV9JsDRL0mPqE1d74kVVDzY1UG%2Bdmdg%3D&reserved=0). PACE helps ensure that figures meet PLOS requirements. To use PACE, you must first register as a user. Registration is free. Then, login and navigate to the UPLOAD tab, where you will find detailed instructions on how to use the tool. If you encounter any issues or have any questions when using PACE, please email PLOS at [figures@plos.org](mailto:figures@plos.org). Please note that Supporting Information files do not need this step.
