# Supplementary material for: Assessment of the transmission blocking activity of antimalarial compounds by membrane feeding assays using natural Plasmodium falciparum gametocyte isolates from West-Africa
Source: PLoS One. 2023 Jul 26;18(7):e0284751. doi: 10.1371/journal.pone.0284751 (PMC10370769; doi:10.1371/journal.pone.0284751)
Supplement: Attachment — Submitted filename: Response to reviewer comments.docx [file pone.0284751.s002.docx]

Dear editor,

Thank you for considering our manuscript ‘Assessment of the transmission blocking activity of antimalarial compounds by membrane feeding assays using natural Plasmodium falciparum gametocyte isolates from West-Africa’ for publication in PLoS ONE. Based on the reviewer and editorial comments, we have improved and in part rewritten our manuscript to bring across the message clearer. Importantly, these are textual changes and no additional data are added to the revised manuscript.

In addition to the comments we received from reviewers that we included below (and address point-by-point), we have highlighted several other sections that required updating based on new published work (e.g. the WHO Malaria Report and a recent article on a similar assay from Mali) to make our manuscript current.’

We would like our funding statement to read as ‘‘This work was supported by grants from the Dutch PDP fund, Medicines for Malaria Venture and Italian cooperation in Burkina Faso. Teun Bousema and Katharine A. Collins are further supported by a European Research Council (ERC) Consolidator Grant to Teun Bousema (ERC-CoG 864180; QUANTUM). The funders had no role in study design, data collection and analysis, decision to publish, or preparation of the manuscript.’

Yours sincerely,

Issiaka Soulama

**Changes in addition to the reviewer comments/editorial requests that are highlighted in the text:**

Title: since we enrolled asymptomatic parasite carriers, we have removed the word patient from the title

Authors: the revision of the manuscript has resulted in a slight change in author order that all authors approve.

Abstract: we included some more specific results in the abstract.

Introduction: we now included a short statement on recent findings that gametocytes can be artemisinin resistant. This gives an extra sense of urgency to our manuscript. We also updated the malaria morbidity and mortality figures with the latest WHO report.

Methods: We added some general text to help the reader appreciate what work was done in Burkina Faso and what work was performed in the Netherlands.

Line 122. We added details on how we transported blood from the site of phlebotomy to the site of culturing/feeding (request reviewer 1)

Figure 1. We updated this figure to provide a comprehensive flow diagram of the different assays we developed (request reviewer 1).

In line 157, we clarified how we decided on the compound concentrations for field testing.

In lines 178-192, we provide additional detail on exactly how the assay was optimized, as requested by the reviewer 1.

In lines 195 and 202, we introduce the abbreviations TB-DMFA and SPORO-DMFA to help the reader understand in the later results how the two assays complement each other.

In line 234, we provide the requested detail on the statistical test used.

In the Results section, we have added small details (highlighted in yellow) to improve clarity without changing any of the results or analyses from the original submission.

The legends to all figures have been improved for clarity.

Discussion 436-454, we have added a description of how our findings compare with findings from a group in Mali who published a manuscript on a similar methodology while our manuscript was under review at PLoS ONE. Their findings complement ours with some relevant differences in findings that we highlight in this revised discussion section.

**Reply to editorial and reviewer comments**

1. Please ensure that your manuscript meets PLOS ONE's style requirements, including those for file naming.

***In our revision, we adhered to these guidelines***

2. Thank you for stating the following financial disclosure:

The funders had no role in study design, data collection and analysis, decision to publish, or preparation of the manuscript.

***In our revision, we have included this statement.***

At this time, please address the following queries:

a) Please clarify the sources of funding (financial or material support) for your study. List the grants or organizations that supported your study, including funding received from your institution. 
b) State what role the funders took in the study. If the funders had no role in your study, please state: “The funders had no role in study design, data collection and analysis, decision to publish, or preparation of the manuscript.”
c) If any authors received a salary from any of your funders, please state which authors and which funders.
d) If you did not receive any funding for this study, please state: “The authors received no specific funding for this work.”

Please include your amended statements within your cover letter; we will change the online submission form on your behalf.

***In our revision, we have included this in the revised manuscript and in the cover letter.***

3. Thank you for stating the following in the Acknowledgments Section of your manuscript:

This work was supported by grants from the Dutch PDP fund, Medicines for Malaria Venture and Italian cooperation in Burkina Faso. The funders had no role in study design, data collection and analysis, decision to publish, or preparation of the manuscript.

We note that you have provided funding information that is not currently declared in your Funding Statement. However, funding information should not appear in the Acknowledgments section or other areas of your manuscript. We will only publish funding information present in the Funding Statement section of the online submission form. 

Please remove any funding-related text from the manuscript and let us know how you would like to update your Funding Statement. Currently, your Funding Statement reads as follows:

**We would like the funding statement to read as**

**‘This work was supported by grants from the Dutch PDP fund, Medicines for Malaria Venture and Italian cooperation in Burkina Faso. Teun Bousema and Katharine A. Collins are further supported by a European Research Council (ERC) Consolidator Grant to Teun Bousema (ERC-CoG 864180; QUANTUM). The funders had no role in study design, data collection and analysis, decision to publish, or preparation of the manuscript**.

We have added this to the cover letter.

Please include your amended statements within your cover letter; we will change the online submission form on your behalf.

4. Please include your tables as part of your main manuscript and remove the individual files. Please note that supplementary tables (should remain/ be uploaded) as separate "supporting information" files

**We have done as instructed**

Reviewers' comments:

Reviewer's Responses to Questions

**Comments to the Author**

1. Is the manuscript technically sound, and do the data support the conclusions?

The manuscript must describe a technically sound piece of scientific research with data that supports the conclusions. Experiments must have been conducted rigorously, with appropriate controls, replication, and sample sizes. The conclusions must be drawn appropriately based on the data presented.

Reviewer #1: Partly

Reviewer #2: Yes

2. Has the statistical analysis been performed appropriately and rigorously?

Reviewer #1: No

Reviewer #2: N/A

**We have improved clarity on the statistical tests we performed. This is now mentioned in the revised methods section and the relevant parts of the results/figure legends.**

3. Have the authors made all data underlying the findings in their manuscript fully available?

The PLOS Data policy requires authors to make all data underlying the findings described in their manuscript fully available without restriction, with rare exception (please refer to the Data Availability Statement in the manuscript PDF file). The data should be provided as part of the manuscript or its supporting information, or deposited to a public repository. For example, in addition to summary statistics, the data points behind means, medians and variance measures should be available. If there are restrictions on publicly sharing data—e.g. participant privacy or use of data from a third party—those must be specified.

 Reviewers' comments:

Reviewer #1: No

Reviewer #2: Yes

**Data have been provided as supplemental file. In a single excel file we provide the exact data that underly the figures.**

4. Is the manuscript presented in an intelligible fashion and written in standard English?

PLOS ONE does not copyedit accepted manuscripts, so the language in submitted articles must be clear, correct, and unambiguous. Any typographical or grammatical errors should be corrected at revision, so please note any specific errors here.

Reviewer #1: Yes

Reviewer #2: No

**Based on this reviewer comment, we have carefully re-written the text and apologize for the typographical and grammatical errors in the initial submission.**

5. Review Comments to the Author

Please use the space provided to explain your answers to the questions above. You may also include additional comments for the author, including concerns about dual publication, research ethics, or publication ethics. (Please upload your review as an attachment if it exceeds 20,000 characters)

Reviewer #1: The authors developed an indirect membrane feeding assay (indirect-MFA), where researchers can test transmission-blocking activity of antimalarial drugs with field gametocytes after 24-hour incubation, and compared IC50 of several drugs between the indirect-MFA and standard membrane feeding assay (SMFA) with laboratory-adapted NF54 parasites (a gold standard method at this moment). While their scientific approach is reasonable, the major results are missing in the current manuscript; i.e., the authors did not submit Table 1. Therefore, this reviewer cannot assess whether their statements are supported by the data. Furthermore, while the development of indirect-MFA is one of major points of this manuscript, the method section is not satisfactory. Thus, it is difficult for other researchers to use the new assay.
**We have taken this comment very seriously and have provided additional detail on our methodology, including exact conditions of blood samples in the feeder. We also added a new Figure 1 that helps explain what assays were used. Our revised manuscript allows readers to replicate findings; the provision of the data underlying the figures further helps in data interpretation.**

Major comments;
1) Please submit Table 1, and show statistical results (e.g., Pearson correlation coefficients and p-values) to support the conclusion; good agreement in IC50 among Gametocyte viability assay, indirect-MFA and indirect-SMFA (in Line 266-268).

**We have clarified our statistical tests. There was confusion in the original manuscript and no correlation coefficients were in fact presented. We apologize for this unclarity that arose from an early draft of a report. Our manuscript is now consistent throughout and includes details on the statistical tests and the cut-off for significance in the methods section and in the figure legends.**

2) Method for indirect-MFA
Please describe the method in detail so that readers can use the assay. More specifically;
During the 24-h incubation
(a) medium; Was it pure RPMI (based on Line 202), or same as NF54 culture medium (including hypoxanthine, HEPES and sodium bicarbonate; Line 158-160), or something else?
(b) container; flask, tube or plate to keep the infected blood
(c) hematocrit; was the blood diluted at a certain ratio, or same ratio of RBC/serum in the original blood?
(d) IC50 was pre-determined to set 0.1x, 1x and 10x IC50 concentrations. But it is not clear how IC50 for each drug was estimated; IC50 in prevalence of infected mosquitoes or that for oocyst density? What assay (and which strain of parasites) was used to determine the value? Did you use a value from publication (if so, cite the paper), or did you use your own data?

**We have now included full details on medium condition (a), the way in which infected blood was stored and how this method was validated (b), the source details and volumes of serum and RPMI (c). Moreover, we describe in detail how we decided on the IC50 estimates, these were based on prior SMFA experiments where oocyst density was used as read-out. We cited the relevant paper, that was from our own group (d)**

For the feed,
(e) composition of feeding samples; If 100% of the liquid part was replaced by a malaria naïve serum after the 24-h incubation, then there was no room to mix the test drug. Was it a mixture of XX%v/v of human serum and XX%v/v of drug solution in RPMI? And what was the hematocrit of final feeding samples?

**Final hematocrit was not determined but we provide an exact procedure, including volumes, in the revised manuscript.**

Minor comments;
3) Can Indirect-MFA check gametocytocidal activity?

**This is a relevant point and also made us realize that indirect MFA was not ideal as terminology. We have now clarified in the introduction, methods and Figure 1 what the different DMFA experiments detect. For instance, the legend to the new figure 1 now reads: ‘Natural gametocyte isolates were used for two distinct assays detecting the overall transmission-blocking activity of compounds by incubating them with gametocyte infected blood for 24 hours (2. TB DMFA) or sporontocidal activity by directly adding compounds to a gametocyte positive blood meal just prior to feeding (1. SPORO-DMFA). ‘**

**The indirect-MFA (now called TB-DMFA) detects the effect of compounds against gametocytes and the sporontocidal effect. We can differentiate between direct anti-sporogony effects and effects that are only apparent upon longer exposure (and are thus likely to be anti-gametocyte).**

Since there was no evaluation for gametocytemia or exflagellation after the 24-h drug treatment, from the lower infection prevalence, we cannot tell whether the drug has sporontocidal activity, gametocytocidal activity, sterilizing gametocytes, or mixture of them. For example, if a drag kills gametocytes during the 24-h incubation (gametocytocidal), the final gametocytemia should be lower than that in the starting blood, but it was not evaluated. On the other hand, a drug may just reduce female fertilization activity (which is technically very difficult to measure) without killing them. In any case, it is impossible to tease out the mechanism of action from the described indirect-MFA method. Please fix all related text in Introduction, Results and Discussion sections.

**We agree and have updated the text accordingly. All amended text related to this comment is highlighted. We have carefully described the extent to which our assay allows discriminating the effects on gametocytes from a sporontocidal effect, as well as limitations in differentiating between gametocyte killing and sterilizing effects.**

4) “overnight” incubation?
People usually think “overnight” means ~10-12-h, not 24-h. To avoid the confusion, please do not use a word of “overnight” throughout the paper.

**We agree and have updated the text accordingly. It was 24 hours; this is now also explained in the figure that depicts the workflow.**

5) Gametocyte viability assays
Based on the method section (Line 161-163), two types of gametocytes (early and late gametocytes) were prepared. But I guess Gametocyte viability assays were performed only using late gametocytes. If so, where did the authors use the “early gametocyte”?
In addition, for clarity, please specify that the assay was done with NF54, such as, we performed full dose response gametocyte viability assays with “NF54” for selected compounds (Line 263-264).

**We apologize for this mistake in the original agree and have updated the text accordingly. Stage III-V gametocytes were used in the gametocyte viability assay, as described in earlier publications.**

6) What is “indirect SMFA”?
In Line 254, a term of “SMFA” is written, but another term “indirect SMFA” is seen in Line 267. Are they the same? If so, please use the same terminology to avoid a confusion. If different, please use different words, e.g., “direct SMFA” and “indirect SMFA”. In addition, I guess “indirect SMFA” means that an assay where NF54 parasites are pre-incubated with a test drug for 24 h before feed, but please clarify.

**We agree with this and have updated the text. Based on this important comment, we have also chosen a different name for our assays: TB-SMFA to describe transmission blocking activity that may be caused by anti-gametocyte or by sporontocidal effects and SPORO-SMFA that is purely detecting anti-sporogony effects by adding the compound of interest directly to the blood meal without an incubation step. We believe that, with our figure 1 and repeated explanations of the differences between these assays, we have improved clarity and satisfied the reviewer.**

7) Bars and error bars
For all figures, please explain what bars (e.g., average, median, geometric mean) and error bars (e.g., sd, sem, 95%CI) mean.
**This has been clarified in the figure legends. All error bars were sd. Some figures are updated based on reviewer comments.**

8) Fig 4
Same as Fig 3, “paired” data points (same donor’s blood were treated with DMSO or drug for 24-h) should be linked. Without the lines, readers cannot interpret the results. For example, the authors conclude that no inhibition by Atovaquone for one isolate (Line 312-313). But if the infection rate for the one isolate changed from ~70% (the highest point in DMSO) to ~30% (the highest point in 100 nM drug), then the conclusion should be different. In addition, a paired test should be used for the statistical analysis (please specify the name of statistical test). Once the authors do so, I’m afraid conclusion may change, e.g., there could be a significant reduction by Methylene blue (and also by DHA). Please revise the related text if needed.
**We have clarified this and provided updated figure. The findings, in terms of statistical significance, did not change: the test was robust and already took into consideration the paired nature of the findings. We do, however, agree that connecting lines are highly valuable for data interpretation.**

9) Typo
Line 173; 10 to the power of 4, not 104

**This was updated**

Line 173, 174 and 176; 30 microliter, not 30 mL

**Updated**

Line 247; to determine the “infection rates”, not “oocyst intensities” (no TRA data in this paper).
**Updated**

Line 307, take out “on”

**Correct, this was updated**

Line 320 and 334; the format of citation is wrong.

**References and all typo’s have been rectified**

Reviewer #2: This is paper describes a method to determine transmission reducing activity of test compounds against P. falciparum isolates. Overall the authors have done an admirable job. Testing field isolates are logistically challenging but very important.

I have comments below to improve the manuscript and make it more transparent to readers.

1) The writing could be polished by a native English user. In several places, the tense is in appropriate and the article is missing. Words such as mosquitoes/mosquitos or transmission blocking/transmission-blocking should be harmonized throughout the manuscript.

**We appreciate this comment and have drastically improved the grammar and style of the revised manuscript. This prompted a complete revision of parts of the manuscript**

2) There is no description of how the transmission blocking experiment was done on 3D7/NF54 gametocytes (i.e. the Indirect-SMFA). I am not sure if the data are original to the study, or referred to published work. Please provide the details of this experiment in the method (if original data), or in the discussion (if from previous studies).
 **This has been clarified. No SMFA was performed as part of this study but the DMFA experiments have been better explained and the names of the assays was updated to improve clarity. We now describe the assays as SPORO-DMFA to detect sporontocidal effects and TB-DMFA to detect general transmission-blocking effects.**

I ask this because it is not clear whether indirect-SMFA is comparable to Indirect-MFA. Did the experiment use enriched gametocytes, and if so, early gametocytes or late gametocytes? Was the treatment also for 24 hours in the same culture medium? Without these details, it’s difficult to know whether the discrepancy in the results of 3D7/NF54 vs field isolates was due to biological (i.e. isolate-to-isolate) differences or the technical aspects of the assays.

3) Line 109: Were the participants treated for malaria after providing 9 ml blood?
**Indeed, donors received treatment. This has been clarified.**

4) Figures 1 & 2 are not clear. The arrow after the 24 hour incubation should point to the mosquito feeding cup.
**We have clarified the figure and now have a single figure that explained the workflow.**

5) Malaria naïve serum used for a) the 24 hr culture medium or b) plasma/RPMI replacement before MFA: was it from an AB blood group donor? Please clarify this in the method section.
**The source of blood (European serum A) was clarified throughout the manuscript**

6) Line 116: “In D0 DMFA” Should this be D1 DMFA?
**The reviewer is correct that this was a mistake, the text has been updated.**

7) Under the method section: Please add statement that, for Indirect DMFA, naïve serum containing the test compound at the test concentration was used to replace the medium before membrane feeding.
**This has been updated**

8) Keywords: Anopheles coluzzii? I thought the experiments used An. gambiae throughout.
**The reviewer is correct. This has been rectified.**

9) Although the term ‘indirect DMFA’ is understandable in the context of this study (lines 351/362), the full acronym is ‘indirect direct membrane feeding’ is rather confusing.. I would suggest just using “indirect MFA” throughout the manuscript.
**As indicated above, we actually renamed our assays to avoid Direct Direct Membrane Feeding Assay and Indirect Direct Membrane Feeding Assay.**
10) Line 245: Please elaborate clearly that these IC50 represents the IC50 of 3D7 gametocytes in the membrane feeding assay. **This has been clarified, with the appropriate references.**
11) Line 245: Please elaborate what ‘duplicate’ means. Does it mean two feeders per isolate?
**This has been clarified; it indeed indicates two feeders**

12) Table 1 was not available to reviewer (i.e. missing). **This was a mistake and has been rectified in the revised manuscript.**

13) It would help reader to harmonize the order of compounds in Figures 5 and 6.
**We have updated the figures and legends.**

14) Line 154: Were dissection also done in plain distilled water? If not, please elaborate whether there is any impact of using water instead of PBS.
**Dissections were done in PBS; this has been clarified.**

15) All analyses were on the infection rate. Because the numbers of oocysts were recorded (line 154), it would be good to analyze the data using the mean oocyst density (i.e. to determine the transmission-reducing activity rather than the transmission-blocking activity). This is probably a more linear/robust readout of % reduction of gametocyte infectivity.
**We have presented all data as % infected mosquitoes. This is the most informative outcome measure for field experiments. While it is true that oocyst density can also be used for mosquito feeding assays with high mosquito infection intensities, these high intensities were not achieved in our study. This has been clarified in the statistical analysis section.**

16) Gametocyte viability assay: did it use early gametocytes or late gametocytes that were described in the parasite culture section?

17) Line 173: 3.5*104: superscript 4?

18) Discussion: A curious minds will want to know why incubation in whole blood for 24 hour led to loss of transmissibility. Would be nice to offer some speculation in the discussion. Was it possible that the parasites were simply eaten/destroyed by white cells?

19) Line 232: It may help to quickly state the effect of these compounds on 3D7 gam transmission (if data/references are available) to provide some mental calibration to understand where the data stand.

20) Line 290: “for during 24 hours at least”
Please soften the claim. There is not strict requirement for this. Although 24 hours is useful, someone might say 20 hours is sufficient.

21) Line 308: please provide reference for “In the absence of a pre-incubation with gametocytes, DHA, MM048 and Methylene Blue on did not affect infectivity of field gametocytes.”

22) Line 334, please reformat the reference.

23) Line 361 “this could explain our result”. Please elaborate further.

I agree that the effect on early gam could explain the lack of inhibition in Indirect-MFA using field isolates. But does this also mean that the blockage of 3D7 by DHA (as mentioned on line 255) was because the experiment was performed using early gametocytes? Readers would want you to help dispel the source of discrepancy between 3D7 and field isolates.

6. PLOS authors have the option to publish the peer review history of their article ([what does this mean?](https://eur02.safelinks.protection.outlook.com/?url=https%3A%2F%2Fjournals.plos.org%2Fplosone%2Fs%2Feditorial-and-peer-review-process%23loc-peer-review-history&data=05%7C01%7CTeun.Bousema%40radboudumc.nl%7C6e216ee993c44a8a006108dafa472ac1%7Cb208fe69471e48c48d87025e9b9a157f%7C1%7C0%7C638097483146554364%7CUnknown%7CTWFpbGZsb3d8eyJWIjoiMC4wLjAwMDAiLCJQIjoiV2luMzIiLCJBTiI6Ik1haWwiLCJXVCI6Mn0%3D%7C2000%7C%7C%7C&sdata=X3XCjPl4huUWkz5V1fABRQtNqRwS3Ic5i3B%2FK%2FpeMDc%3D&reserved=0)). If published, this will include your full peer review and any attached files.
